# P-2. Ceftaroline Monotherapy Versus Combination Therapy for Persistent Methicillin-Resistant Staphylococcus aureus (MRSA) Bacteremia: A Retrospective Cohort Study

**DOI:** 10.1093/ofid/ofaf695.233

**Published:** 2026-01-11

**Authors:** Ali Althubyani, Milton O Kodom, Dana J Holger

**Affiliations:** Nova Southeastern University, Davie, FL; Nova Southeastern University/Memorial Healthcare System, Fort Lauderdale, FL; Nova Southeastern University, Davie, FL

## Abstract

**Background:**

Persistent methicillin-resistant *Staphylococcus aureus* (MRSA) bacteremia is associated with high mortality. Ceftaroline monotherapy is a promising treatment, and combining ceftaroline with daptomycin or vancomycin has shown in vitro synergistic effects that may enhance clinical outcomes. This real-world study compares the effectiveness of ceftaroline monotherapy versus combination therapy in treating persistent MRSA bacteremia.

**Figure:**
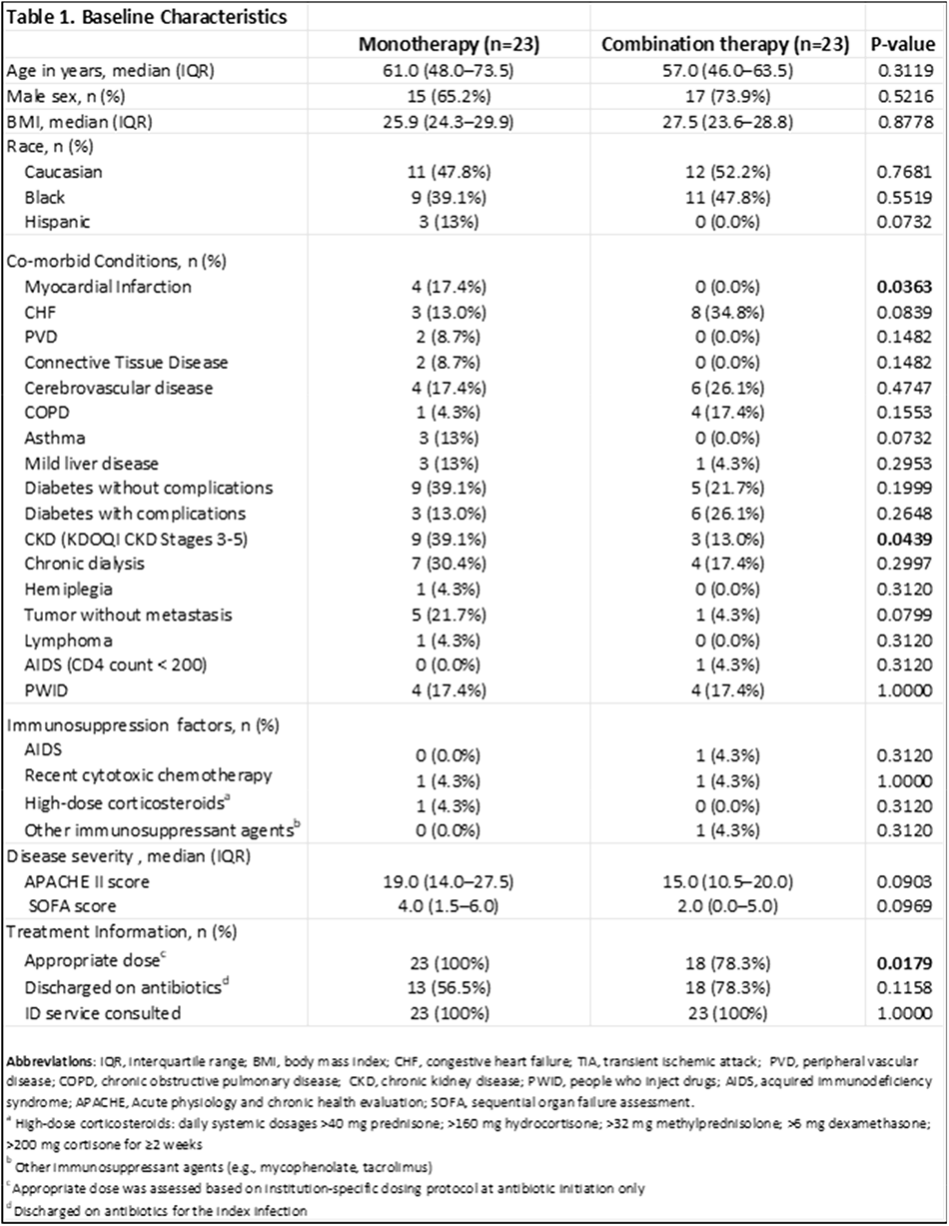

**Figure:**
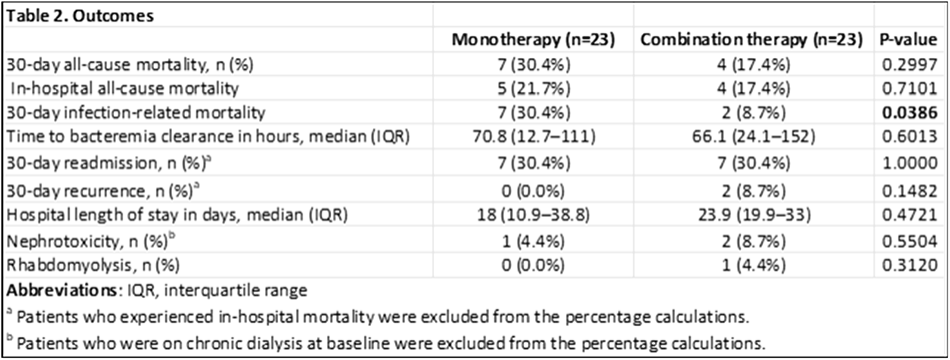

**Methods:**

This multicenter, retrospective cohort study included patients with persistent MRSA bacteremia (i.e., positive blood cultures after 48 hours of anti-MRSA therapy) from five hospitals across the Memorial Health Care System between January 2019 and September 2023. Included patients received ≥48 hours of ceftaroline monotherapy or combination therapy with ceftaroline plus either daptomycin or vancomycin. Exclusions were lack of repeat blood cultures, polymicrobial bacteremia, pregnancy, or incarceration. The primary outcome was 30-day all-cause mortality; secondary outcomes included in-hospital mortality, 30-day infection-related mortality, time to bacteremia clearance, 30-day readmission, 30-day recurrence, and adverse events. Logistic regression identified predictors of 30-day mortality.

**Figure:**
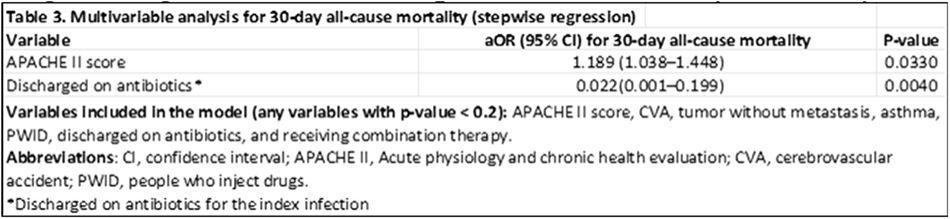

**Results:**

Among 46 adults (23 per arm), baseline demographics and severity scores were similar except for higher rates of myocardial infarction (17% vs 0%, p=0.036) and CKD (39% vs 13%, p=0.044) in the monotherapy group; appropriate dosing was more frequent with monotherapy (100% vs 78%, p=0.018). 30-day all-cause mortality was lower with combination therapy (17% vs 30%) but not statistically significant (p=0.299), whereas infection-related mortality was significantly reduced (9% vs 30%, p=0.039). For all other secondary outcomes, no differences were observed. In multivariable analysis, higher APACHE II score (aOR 1.53, 95% CI 1.12–2.56; p=0.039) and discharge on antibiotics (aOR 0.004, 95% CI < 0.001–0.097; p=0.012) predicted 30-day mortality.

**Conclusion:**

Combination therapy with ceftaroline plus daptomycin or vancomycin may reduce infection-related mortality, with a trend toward lower 30-day mortality. These findings suggest potential clinical benefit and warrant further study in a larger sample.

**Disclosures:**

All Authors: No reported disclosures

